# A significant vascular variant in oncologic pancreaticoduodenectomy: the arc of Buhler

**DOI:** 10.1186/s40792-022-01387-9

**Published:** 2022-03-02

**Authors:** L. Schumacher, H. C. Albrecht, S. Gretschel

**Affiliations:** Faculty of Health Brandenburg, Brandenburg Medical School, Department of General, Visceral, Thoracic and Vascular Surgery, University Hospital Neuruppin, Fehrbelliner Strasse 38, 16816 Neuruppin, Germany

**Keywords:** Arc of Buhler (AOB), Arterio-arterial anastomosis (shunt), Superior mesenteric artery (SMA), Celiac trunk (CA), Gastroduodenal artery (GDA), Pancreaticoduodenectomy

## Abstract

**Background:**

The arc of Buhler (AOB), a rare anastomosis connecting the superior mesenteric artery (SMA) to the celiac trunk (CA), was found in a patient suffering from an adenocarcinoma of the pancreatic head.

**Case presentation:**

Oncologic pancreaticoduodenectomy required resection of the AOB to achieve complete tumor removal. After an uneventful clinical course in the first days, the patient suffered a severe complication. Due to ischemia of the stomach and spleen, complete resection of the stomach, spleen, and remaining pancreas had to be performed.

**Conclusions:**

The hemodynamic impact of this arterial variant has been discussed mainly for liver perfusion, which remained intact at all times in our case. Because of the serious obstacles mentioned above, we strongly recommend that the presence of AOB be considered in preoperative diagnosis and preservation when possible. If the AOB is likely to be ligated, stenosis of the SMA or CA should be excluded and resolved before surgery.

## Background

Variants and anomalies of the abdominal visceral vessels are frequently found in patients [1]. However, an arterio-arterial anastomosis between the SMA and the CA, called the arc of Buhler (AOB), is present in only less than 3% of cases [2]. The first description of this variant dates from 1904, identified by Bühler and Tandler [3]. The AOB is thought to be a relic of ventral longitudinal anastomosis of the 11th and 13th intersegmental arteries during embryogenesis. In healthy, asymptomatic individuals, this collateral vessel is presumed to fulfill a significant hemodynamic role in approximately half of the cases [4]. However, in most reported cases, total or near-total stenosis of the CA is also found, making perfusion of the liver, stomach, and spleen potentially AOB-dependent [1]. Therefore, concerns have been raised about AOB in upper GI surgery. To our knowledge, we hereby report the first case involving the resection of the AOB during abdominal surgery.

## Case presentation

A 66-year-old patient was admitted to the hospital because of painless jaundice and pathologic elevated cholestasis parameters. Conventional sonography revealed a malignancy-suspect tumor of the pancreatic head with dilatation of the common bile duct. Subsequently, endoscopic retrograde cholangiopancreatography was conducted with papillotomy and stenting of the common bile duct. No malignant cells were detected in the biopsies taken, although the suspected diagnosis of a malignant pancreatic head tumor was supported by endoscopic ultrasound. Contrast-enhanced computed tomography of the thorax and abdomen showed no metastases, and therefore curatively intended pancreaticoduodenectomy was evaluated.

**Fig. 1:**
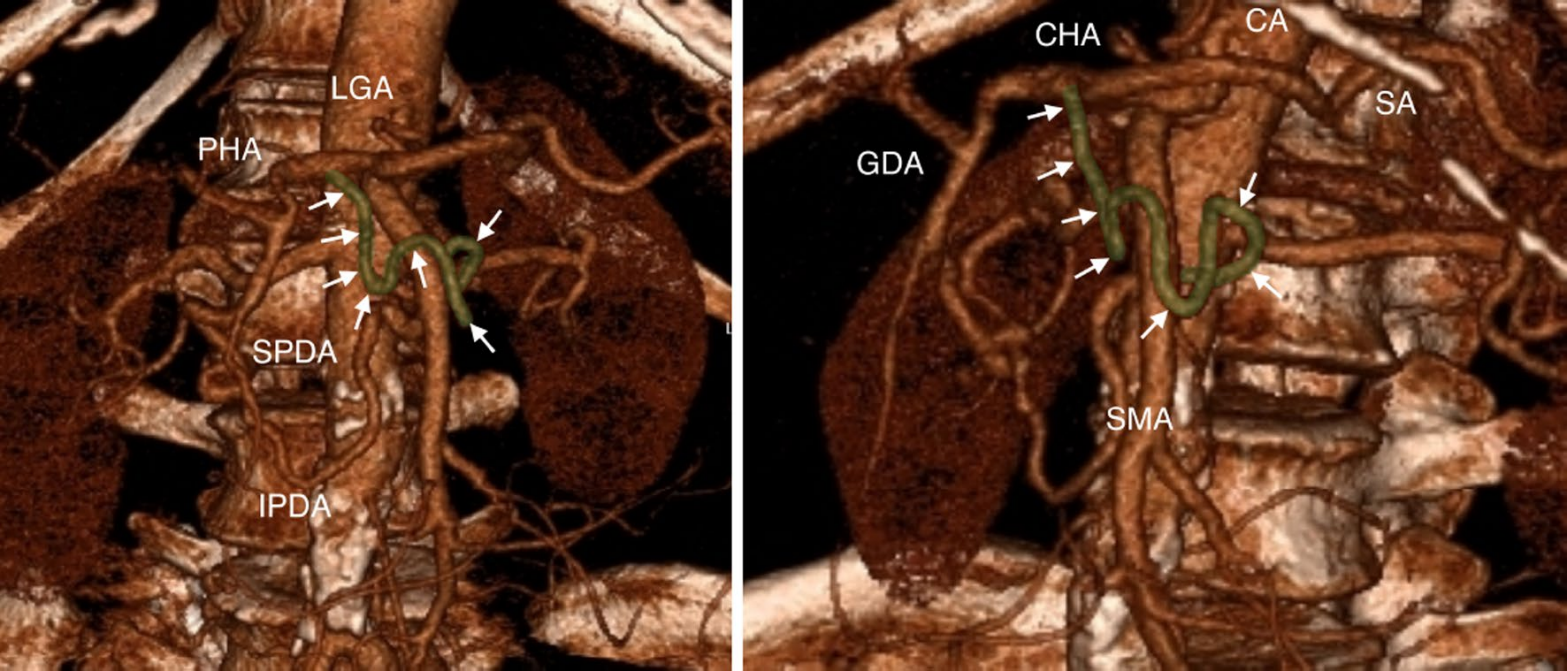
Identification of the arc of Buhler (*white arrowheads, highlighted green*) before surgery by a three-dimensional reconstructed computed tomography. *CA* celiac artery, *CHA* common hepatic artery, *SA* splenic artery, *GDA* gastroduodenal artery, *SMA* superior mesenteric artery, *LGA* left gastric artery, *PHA* proper hepatic artery, *SPDA* superior pancreaticoduodenal arteries, *IPDA* inferior pancreaticoduodenal arteries

3D reconstruction of the upper abdominal vessels from CT data was performed to plan the surgical treatment. There was no evidence of local irresectability, but an arterio-arterial shunt was discovered originating from the dorsal part of the SMA and leading to the common hepatic artery (CHA), just after its branching from the CA (Fig. 1). The vessel joined the SMA and celiac trunk independently of the common arterial anastomosis, therefore it was considered to be the AOB. Furthermore, the celiac trunk appeared to be stenotic. After critical discussion with our radiology department, preoperative stent implantation was not performed as the stenosis of the CA was considered marginal.

A median arcuate ligament compression syndrome (Dunbar syndrome) could also have been the cause of the described stenosis. Because of the leading oncologic diagnosis, no further investigation of asymptomatic CA stenosis was performed.

**Fig. 2:**
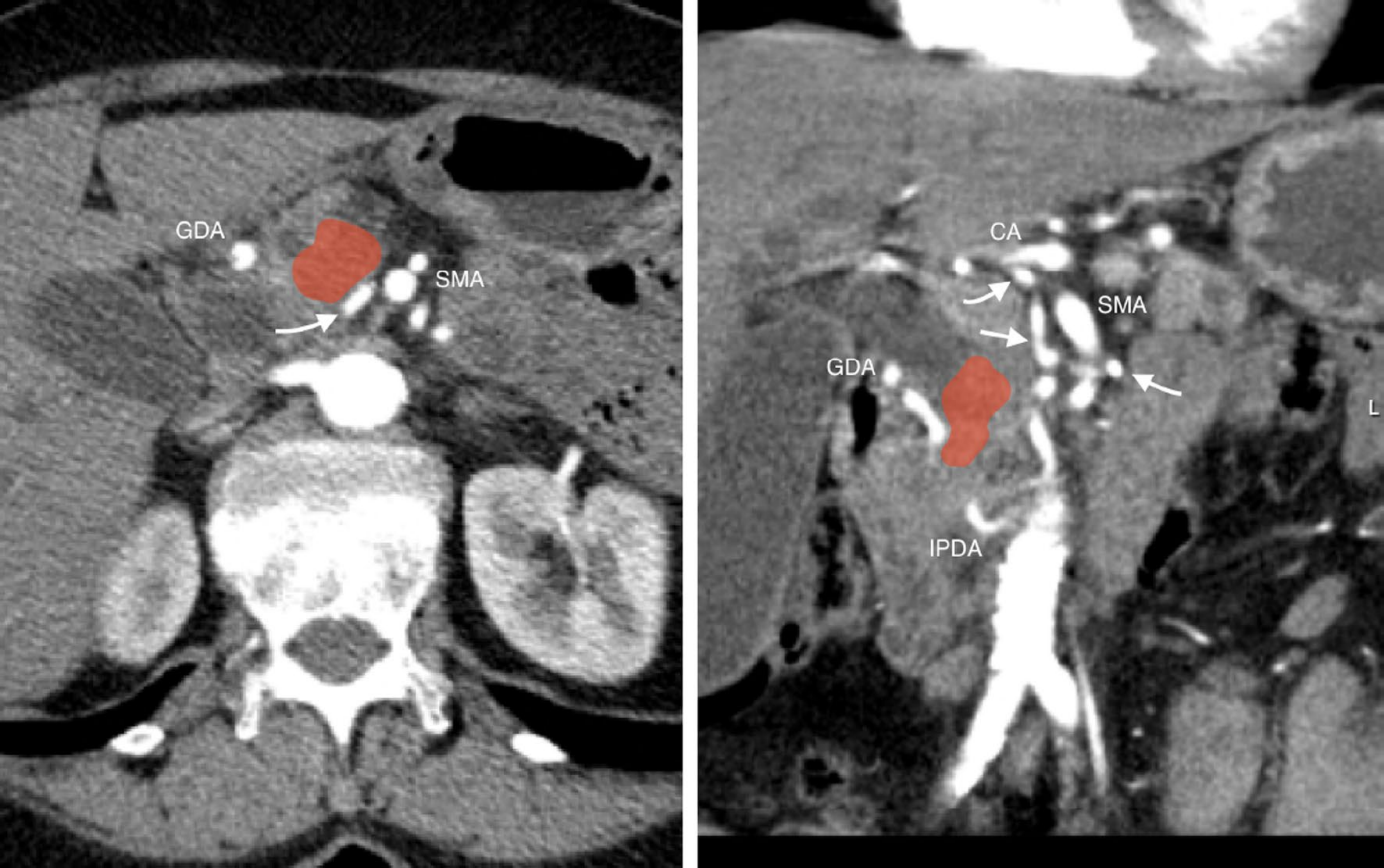
Computed tomography image showing the tumor of the pancreatic head (*highlighted red*) in proximity with the the arc of Buhler (*white arrowheads*) and the *GDA* gastroduodenal artery. *SMA* superior mesenteric artery, *CA* celiac artery, *PDA* inferior pancreaticoduodenal arteries

The AOB showed close proximity to the tumor and possible tumor infiltration (Fig. 2). We planned pancreaticoduodenectomy with the intention of preserving the AOB if technically and oncologically feasible.

Intraoperatively, we found a locally advanced tumor with regional inflammatory changes. After mobilization of the duodenum and pancreatic head, the gastroduodenal artery (GDA) was ligated.

As the dissection progressed toward the pancreatic body, the AOB was identified cranial to the tumor.

Further caudal tumor infiltration could not be excluded, making preservation of the shunt for oncologic resection impossible. After clamping the AOB close to its termination at the CHA, there was no evidence of ischemia of the liver or stomach. This was verified by Doppler ultrasonography. The AOB was resected. The proper hepatic artery (PHA) showed a steady pulse at all times. Pancreatic anastomosis was constructed as a pancreaticogastrostomy. Single-loop reconstruction was performed for bilio-jejunal and gastro-jejunal anastomosis.

The early postoperative course was uneventful. On postoperative day 2, a planned gastroscopy showed no evidence of anastomotic insufficiency or ischemia. Having already received regular meals, the patient developed abdominal pain and elevated infection laboratory markers on postoperative day 9. An immediate CT scan of the abdomen indicated an insufficient pancreatic–gastric anastomosis. Emergency relaparotomy revealed gastric ischemia with insufficiency of the pancreatogastrostomy and gastrojejunostomy and necrosis of the remnant pancreas. In addition, the splenic artery (SA) showed a relatively small diameter and thrombosis. We performed gastrectomy, splenectomy, resection of the remaining pancreatic and reconstruction as esophago-jejunostomy. The patient stabilized quickly with ICU-treatment. After drainage of the pleural effusion, treatment of wound dehiscence, and wound infection, the patient was discharged from the hospital on postoperative day 44.

The result of histologic examination confirmed complete resection of a 37-mm, moderately differentiated ductal adenocarcinoma of the pancreas with infiltration of the duodenum and common bile duct (TNM: pT2, pN 1 (3/21), L 1, Pn 1, G 2, R 0). There was no evidence of tumor infiltration of the resected arterial shunt.

## Discussion

For the successful performance of surgical procedures in the upper gastrointestinal tract, it is important to recognize and consider vascular anomalies, especially in pancreatic surgery [5]. Although the AOB was first described in 1904, the rare occurrence of this anastomotic artery and the relatively low prevalence of pancreatic disease make an implication rare. A recent review showed that only 53 cases of the AOB have been described [1]. Considering the patient from our clinic and a recent case report, the number has increased to 55 [6].

Using PubMed for a literature review with the keywords “pancreaticoduodenectomy” and “arc of buhler”, we could identify only four cases involving pancreaticoduodenectomy and the coincidental presence of an AOB (Table 1). In all four cases the AOB was preserved during surgery. In two cases, the vessel was identified before surgery so that a planned approach could be undertaken [5, 6]. These patients suffered from adenocarcinomas of the ampulla of Vater, which had different locoregional conditions compared with the case reported here. One author reported identifying the shunt postoperatively because of bleeding on day 5 after pancreaticoduodenectomy of a ductal adenocarcinoma [7]. McCracken reported an accidental discovery of AOB during surgery [8]. The patient showed stenoses of the CA and SMA. Due to the coincidental presence of the AOB, no stent or arterial bypass procedure was required after GDA resection.

**Table 1:** Previous reports of surgical pancreaticoduodenectomy in patients with a present arc of Buhler

Author (year)	Condition	Surgical treatment	Postoperative course
Templin (2020) [6]	Adenocarcinoma, papilla vateri T3 N2 (7/13) M0	Pancreaticoduodenectomy, preoperative identification of the AOB by digital 3D reconstruction	Mild cholangitis, no further complications
McCracken (2018) [8]	Intraductal papillary mucinous neoplasm, pancreatic head (suspected adenocarcinoma)	Pancreaticoduodenectomy, intraoperative AOB detection by angiography	TRANSIENT transaminitis day 2, discharged day 7. Brief rehospitalization due to Klebsiella bacteremia
Kageyama (2016) [5]	Adenocarcinoma, ampulla of Vater T? N0 M0	Pancreaticoduodenectomy preoperative identification by computer tomography	uneventful, discharged day 14
Ochoa (2016) [7]	Pancreatic ductal adenocarcinoma T3 N0 M0	Pancreaticoduodenectomy, AOB identified after surgery due to complications	Intraluminal bleeding on day 5—> IR intervention. Possible partial left hepatic lobe infarct, bacteremia, discharged day 19

**Fig. 3:**
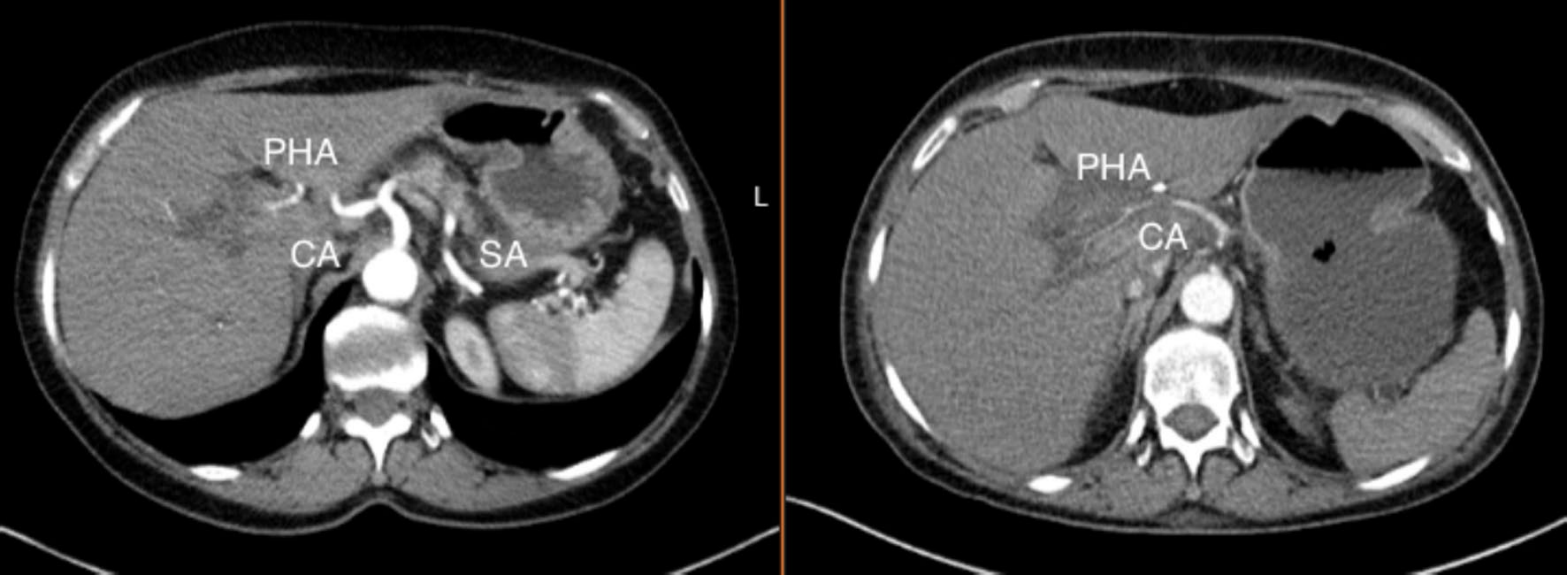
Comparison of the upper GI visceral arteries before and after resection of the AOB. *CA* celiac artery, *PHA* proper hepatic artery and *SA* splenic artery

The discussion in the case reports above emphasized the importance of AOB preservation for liver perfusion. This results from the frequently associated CA stenosis, especially when no other collateral hepatic artery arises from the SMA. In contrast, our patient did not show signs of liver malperfusion at any time; however, major complications from ischemia of the stomach and spleen occurred. The vascular reconstruction of the postoperative CT scan (postoperative day 9) revealed a tremendous loss of vascularization regarding the CA (Fig. 3). Compared to the liver, under normal circumstances the stomach has a distinct blood supply consisting of more than four major arteries. The right gastroepiploic artery arises from the gastroduodenal artery (GDA) and receives regular collateral blood flow through the SMA from functional anastomoses between the pancreaticoduodenal arteries. During pancreaticoduodenectomy, ligation and transection of the GDA and right gastroepiploic artery is mandatory. After dissection of the latter vessels and the AOB, our patient developed compromised blood supply to the stomach and spleen. This gastric ischemia was not evident on staged gastroscopy on postoperative day 2. Thus, we are left to speculate whether and to what extent CA stenting could have prevented ischemia of the stomach, spleen, and pancreatic remnant at postoperative day 9. Knowing the further course, retrospectively the evaluation of such an intervention would have been useful both preoperatively and early postoperatively.

## Conclusion

In the presence of an AOB, caution should be exercised during pancreatic or hepatobiliary surgery. Preoperative angioplasty and stenting should be considered if there is evidence of CA or SMA stenosis. In circumstances requiring AOB resection, liver and gastric perfusion should be critically assessed. Surgeons should be prepared to extend the abdominal resection and possibly perform further vascular reconstruction.

## Data Availability

All data generated or analyzed during this study are included in this published article. Further material and information on this case report are available from the corresponding author on reasonable request.

## Abbreviations

AOB: The arc of Buhler
SMA: Superior mesenteric artery
CA: Celiac trunk
GDA: Gastroduodenal artery
GI: Gastrointestinal
CT: Computer tomography
CHA: Common hepatic artery
PHA: Proper hepatic artery
LA: Splenic artery
ICU: Intensive care unit
